# Efficacy and Safety of Different Bioactive Coils in Intracranial Aneurysm Interventional Treatment, a Systematic Review and Bayesian Network Meta-Analysis

**DOI:** 10.3390/brainsci12081062

**Published:** 2022-08-10

**Authors:** Jie Zhang, Guannan Jiang, Zhaoming Song, Wei Cheng, Wenxue Wu, Zhouqing Chen, Zhong Wang, Wanchun You, Gang Chen

**Affiliations:** Department of Neurosurgery & Brain and Nerve Research Laboratory, The First Affiliated Hospital of Soochow University, 188 Shizi Street, Suzhou 215006, China

**Keywords:** aneurysm, bioactive coils, interventional treatment, efficacy, safety

## Abstract

Background: Bioactive coils have been used for nearly 20 years to improve aneurysm treatments. Previous studies are inadequate for comparing the efficacy and safety between different coils. The aim of this study was to investigate the safety and efficacy of different coils by comparing the percentage of people with different modified Raymond scale grades, re-rupture rates, and mortality in patients with intracranial aneurysms embolized with different coils. Method: Randomized controlled trials (RCTs) containing coils for aneurysm interventional treatment were collected from Web of Science, PubMed, and the Cochrane Library up to December 2021. Bayesian network meta-analysis with a randomized or fixed model was performed to compare the efficacy and safety among different bioactive coils and bare platinum coils. Results: We pooled 3362 patients from eight RCTs. No significant differences were found between coils in the proportion of patients with a three-grade classification assessed with the modified Raymond scale immediately after surgery. Hydrogel coils did not show a significant difference in the percentage of patients with a modified Raymond scale grade I postoperatively compared with bare platinum coils (OR, −0.1080; 95% CI, −0.4201–0.2423), but at follow-up, the percentage of patients with modified Raymond scale grade I was significantly higher with hydrogel coils than with bare platinum coils (OR, 0.4957; 95% CI, 0.0060–0.9442). There were no statistical differences between these four coils in terms of aneurysm rupture or re-rupture rate and mortality. Conclusion: Though there was no significant difference in the embolization effect between the several coils in the postoperative period, complete embolization was more likely to be achieved with hydrogel coils compared to bare platinum coils at follow-up. There were no significant differences in safety between the several coil materials.

## 1. Introduction

Intracranial aneurysm is a common cerebrovascular disease, which frequently occurs in middle-aged and elderly people [1]. It generally has an insidious process and once it ruptures, it has a high mortality and disability rate [2]. Currently, aneurysms are treated by endovascular intervention and microsurgical surgery [3]. Microsurgery is effective in treating intracranial aneurysms but is highly invasive and has many complications [4]. Compared with microsurgery, endovascular interventions have less trauma, faster postoperative recovery, and fewer complications [5]. It has better therapeutic effects for elderly patients who cannot tolerate surgical treatment.

Bare platinum coils were the first interventional coils to be used in endovascular interventions for the treatment of aneurysms. In contrast to surgical clipping, which visually assesses the effect of clipping, endovascular interventions generally assess the size of the residual aneurysm lumen after embolization by imaging. Since the first use by Guglielmi et al. in 1990, the embolization rate of bare platinum coils has been suboptimal [6,7]. To reduce the recanalization rate of aneurysms, bioactive coils were invented, which include cerecyte coils [8], hydrogel coils [9,10], and matrix coils [11]. Cerecyte coils have polyglycolic acid inside [8]; as the name suggests, hydrogel coils are covered hydrogels [9,10]; and matrix coils are polyglycolic/polylactic acid (PGLA)-encapsulated [11]. The addition of these bioactive materials was expected to accelerate fibrosis and endothelial neogenesis in aneurysms and reduce the risk of recurrence.

These bioactive coils have been in clinical use for over 20 years. Some previous studies have shown that the use of bioactive coils may improve embolization compared to bare platinum coils at follow-up. However, some have found no significance, and the conclusions of improvement in recurrence rates are not consistent [12,13,14]. In total, comparisons of efficacy and safety between cerecyte coils, hydrogel coils, matrix coils, and bare platinum coils are not yet adequate. In order to analyze which interventional coils have better embolic efficacy and safety, we performed a Bayesian network meta-analysis to analyze the differences in efficacy and safety of the different embolic materials.

## 2. Materials and Methods

### 2.1. Literature Search

In our study, we used an appropriate search method based on PRISMA guidelines to screen eligible randomized controlled trails on endovascular interventions with coils coated with different materials for the treatment of patients with intracranial aneurysms in 3 literature databases: Web of Science, PubMed, and Cochrane library. The publication dates of the included papers were up to December 2021. The search was performed in both databases following the keywords: (“aneurysm”) AND (“coil” OR “bare platinum coil” OR “cerecyte coil” OR “hydrogel coil” OR “matrix coil”) AND (“randomized controlled trail” OR “RCT” OR “randomized controlled”). In addition, we searched for additional records through manual web searches, manual reference lists screening, and the screening of relevant studies suggested by the databases above.

### 2.2. Inclusion and Exclusion Criteria

Included studies were screened according to the following criteria: (1) randomized controlled trails (RCT) including patients diagnosed with intracranial ruptured or unruptured aneurysm; (2) each article must contain at least 2 kinds of coils, including bare platinum coils, cerecyte coils, hydrogel coils, and matrix coils; (3) each article must contain at least 1 included outcome indicator, such as a percentage of the modified Raymond scale grade I to III patients at postoperative and follow-up, rupture or re-rupture rate, and mortality; and (4) data must be available in publications. Articles were excluded based on the following criteria: (1) papers containing duplicate RCT registration records or patients were excluded and only 1 paper was retained; (2) protocols, meta-analysis, post-hoc analyses studies or relevant articles without data. The whole process was plotted as a PRISMA flow diagram.

### 2.3. Data Extraction

Data on first author, the year of publication, study region, included population, follow-up time, sex ratio, the percentage of the modified Raymond scale grade I to III patients at postoperative and follow-up stages, rupture or re-rupture rate, and mortality were extracted by 2 authors independently. Then data was checked and merged with a third author.

### 2.4. Quality Assessment

The quality assessment of the screened articles was conducted by using the risk of bias assessment tool from the Cochrane Collaboration, RoB 2 tool, to assess the quality of the included articles [15]. A third reviewer was introduced when the twos reviewers disagreed about the process of quality assessment and discussed with the first two to obtain conclusions about the assessment of article quality.

### 2.5. Statistical Analysis

In this study, we followed the Bayesian network construction method and performed a network meta-analysis using R4.0.3 software and GEMTC R-package following the PRISMA guidelines [16]. The odds ratio (OR) and the corresponding 95% confidence interval (CI) were used as valid indicators for this analysis. We first examined each indicator for heterogeneity and used chi-square q-test and *I*^2^ statistics to assess heterogeneity across researchers. If *p* < 0.05 or *I*^2^ > 50% showed significant heterogeneity, a random-effects model was used; conversely, heterogeneity was low, a fixed-effects model was used. The results of the network meta-analysis contained both direct and indirect comparisons, which were presented in the form of forest plots. When indirect evidence was present in the data, we analyzed consistency in that network. To assess consistency, we evaluate the differences between direct evidence, indirect evidence, and pooled network evidence present in each closed loop by node-splitting methods [17].

After that, we created ranked line graphs for each indicator to assess which treatment had the greatest impact on that indicator. League tables were also constructed, with ORs (columns vs. row) for comparisons between 2 treatments below the diagonal and effect sizes plotted above the diagonal. The surface under the cumulative ranking curve (SUCRA) was calculated from the treatment level, and its value was also embedded in the diagonal of the league table [18]. Higher SUCRA values indicated a higher incidence of the outcome.

## 3. Results

### 3.1. Study Characteristics

**Figure 1:**
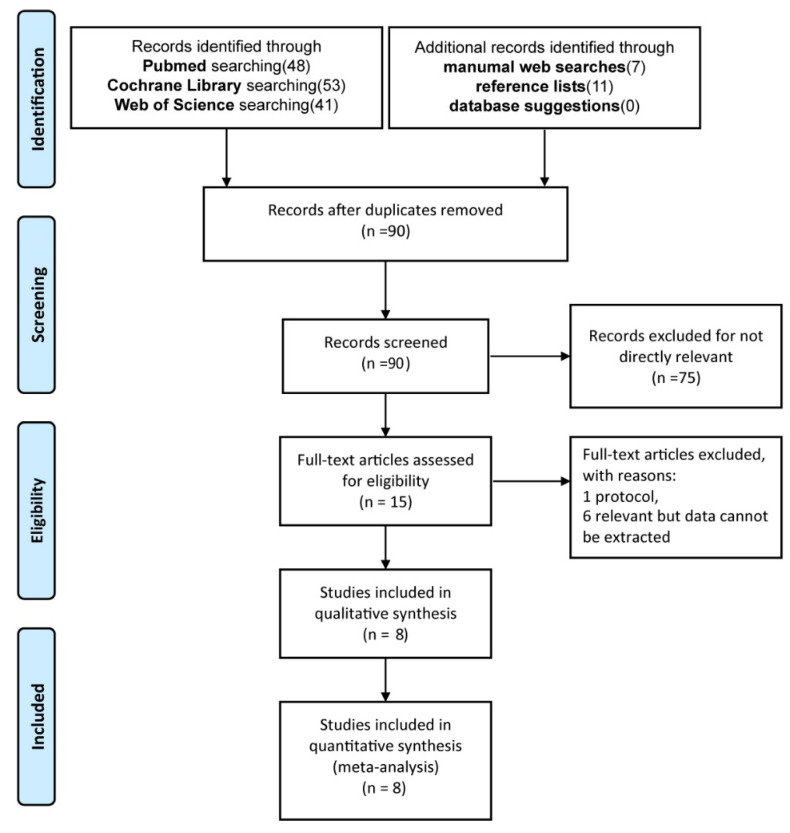
Flow diagram of the literature search and study selection.

We extracted 41 papers from Web of Science, 47 papers from PubMed, and 53 papers from the Cochrane library based on the keywords described in the method. We found seven additional studies by manual web search and eleven additional records by reference list screening. By manual comparison, we removed the duplicates and ultimately obtained 90 papers by preliminary screening and merging. Seventy-five of these were excluded because the titles and abstracts were not directly related to our research objectives. The remaining 15 papers were read and screened in full. Finally, one protocol and six articles with unavailable data were excluded. In total, we included eight papers containing two articles on cerecyte coils [19,20], five articles on hydrogel coils [10,21,22,23,24], and one article on matrix coils [25]. The whole screening process is shown in Figure 1. In Table 1, we summarize the characteristics of the eight included papers. Of the eight included articles, the article by Martin et al. [19] had a smaller proportion of female patients. There was no significant difference in the mean age of the overall included patients. In terms of follow-up time, the article by J. Raymond et al. [21] had a follow-up time of 1 month, while the rest of the literature was greater than or equal to 6 months.

### 3.2. Quality Assessments

**Figure 2:**
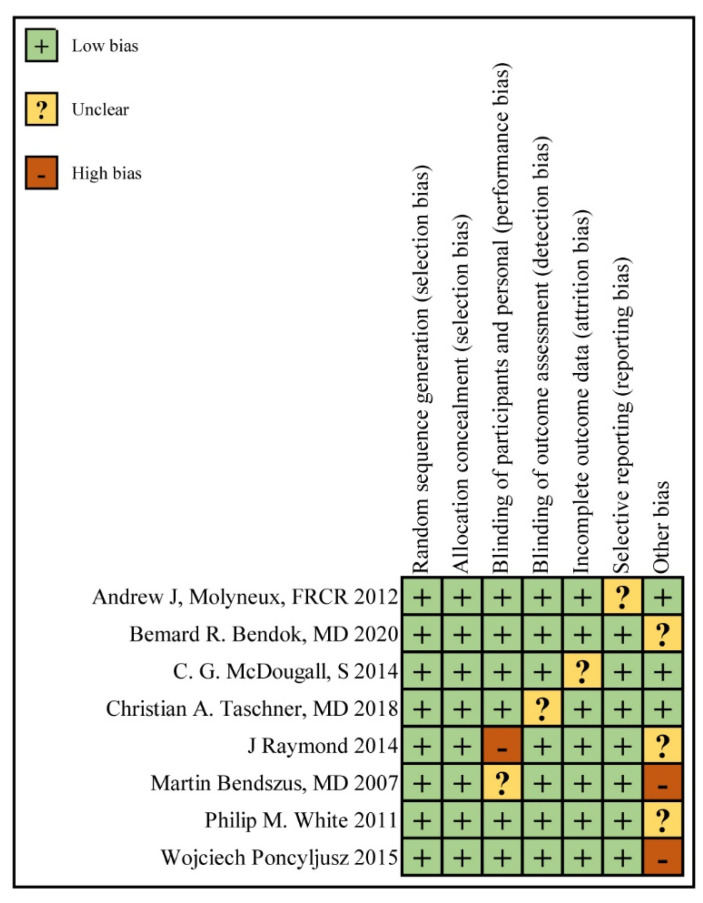
Risk of bias summary: review of authors’ judgements about each risk of bias item for each included study (Martin et al. [18], Andrew et al. [19], White et al. [22], McDougall et al. [24], Raymond et al. [20], Wojciech et al. [21], Christian et al. [23], Bernard et al. [10]).

The quality assessment of the included RCTs is presented in Figure 2. The quality of the included publications was satisfactory. The main risk of bias that could affect the quality of the articles occurred in the double-blind control of the subjects in research by Raymond et al. [21]. Other serious biases were the small sample size, with 110 people included in article by Martin et al. [19] and 96 people included in article by Wojciech et al. [22], which differed from the inclusion numbers in the other articles.

### 3.3. Network Meta-Analysis

We analyzed and generated network plots based on data from previous articles, which are presented in the Supplementary Material Figure S1. The size of the circles and the width of the line segments represent the number of patients and articles included in the paired material, respectively. The Markov chain fitting process for all meta-analyses is shown in Supplementary Material Figure S2. With the trace plot on the left and the density plot on the right, it can be determined that the model converges satisfactorily and the curves tend to be normally distributed in the density plot [26].

**Figure 3:**
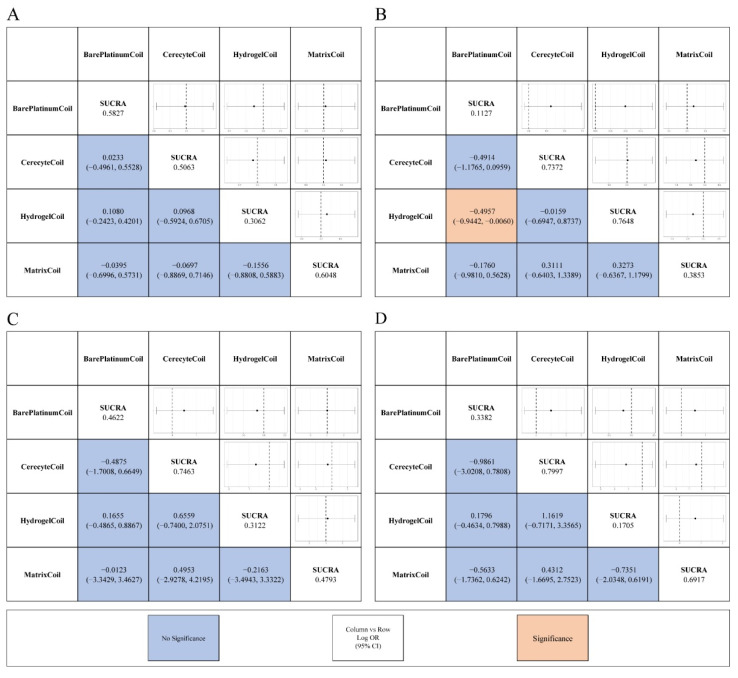
League tables for the outcomes of efficacy and safety generated using fixed or random effect models. (**A**) Percentage of postoperative patients with modified Raymond scale grade I; (**B**) Percentage of patients with modified Raymond scale grade I at follow-up; (**C**) Rates of intraoperative aneurysm rupture or re-rupture; (**D**) Mortality rate.

Firstly, we performed a network meta-analysis to examine the differences in the proportion of patients with modified Raymond scale grade I to III, aneurysm rupture or re-rupture rates, and mortality at postoperative and follow-up stages. In terms of assessing the effectiveness of embolization therapy, no statistically significant differences were found between intervention groups in the proportion of patients with a modified Raymond scale grade I assessed immediately after surgery (Figure 3a). There was also no significant difference in the proportion of patients with modified Raymond scale grade II and III (Supplementary Material, Figure S3a,b).

Although no significant improvement was found for hydrogel coils (OR, −0.1080; 95% CI, −0.4201–0.2423) compared with bare platinum coils in terms of the proportion of patients with modified Raymond scale grade I evaluated immediately after surgery, there was a significant increase in modified Raymond scale grade I at follow-up for patients with hydrogel coils (OR, 0.4957; 95% CI, 0.0060–0.9442) compared with bare platinum coils (Figure 3A,B). For the percentage of patients with modified Raymond scale grade II and III at follow-up, differences could not be detected between the four coil materials (Supplementary Material, Figure S3c,d).

Regarding the safety aspects of coil use, we did not find differences between these four coils in terms of intraoperative aneurysm rupture or re-rupture rates or mortality (Figure 3C,D).

### 3.4. SUCRA and Rank Probability

**Figure 4:**
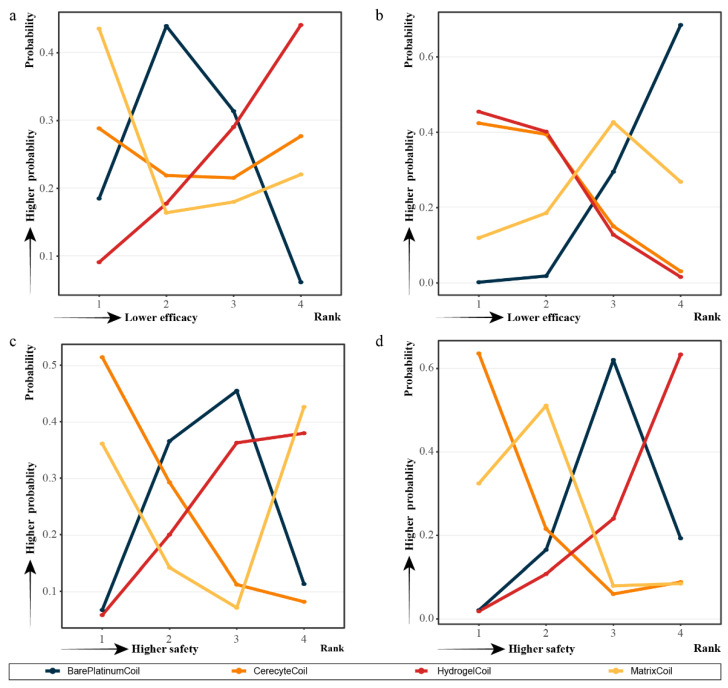
Probability ranks for outcomes of the efficacy and safety generated using fixed or random effect models. (**a**) Percentage of postoperative patients with modified Raymond scale grade I; **(b**) Percentage of patients with modified Raymond scale grade I at follow-up; (**c**) Rates of intraoperative aneurysm rupture or re-rupture; (**d**) Mortality rate.

Based on pairwise analysis in the network, not every coil showed statistically significant differences from the other coils at each indicator. We analyzed the effect of each material coil on each indicator based on multiple inferences by calculating the SUCRA value (Figure 3, Supplementary Material Figure S3) and probability ranking (Figure 4, Supplementary Material Figure S4) for each indicator.

For the evaluation of efficacy immediately after surgery, the use of the matrix coil (SUCRA, 0.6048) had the highest likelihood of obtaining the highest percentage of patients with a modified Raymond scale grade I. The hydrogel coil (SUCRA, 0.3062) ranked the worst in terms of the percentage of patients with a modified Raymond scale grade I after surgery compared to the other coils.

However, at follow-up, the hydrogel coil (SUCRA, 0.7648) was the most likely to have the highest proportion of patients with modified Raymond scale grade I. In addition, the cerecyte coil (SUCRA, 0.5758) and the bare platinum coil (SUCRA, 0.8918) were the most likely to have the highest percentage of patients with modified Raymond scale grade II at postoperative and follow-up, respectively. In contrast, patients treated with hydrogel coils (SUCRA, 0.6183) and matrix coils (SUCRA, 0.7393) were most likely to have the highest proportion of patients with modified Raymond scale grade III postoperatively.

In terms of safety, the cerecyte coils had a higher likelihood of aneurysm rupture or re-rupture (SUCRA, 0.7463) and patient mortality (SUCRA, 0.7997), and the cumulative probability ranking plots showed that they had the highest probability of causing safety risks. Hydrogel coils showed the relatively lowest probability of intraoperative aneurysm rupture or re-rupture (SUCRA, 0.3122) and mortality (SUCRA, 0.1705), while the bare platinum coils (SUCRA, 0.1943) had the lowest mortality rate, although all of these did not differ significantly from the other coils.

### 3.5. Heterogeneity Analysis

**Figure 5:**
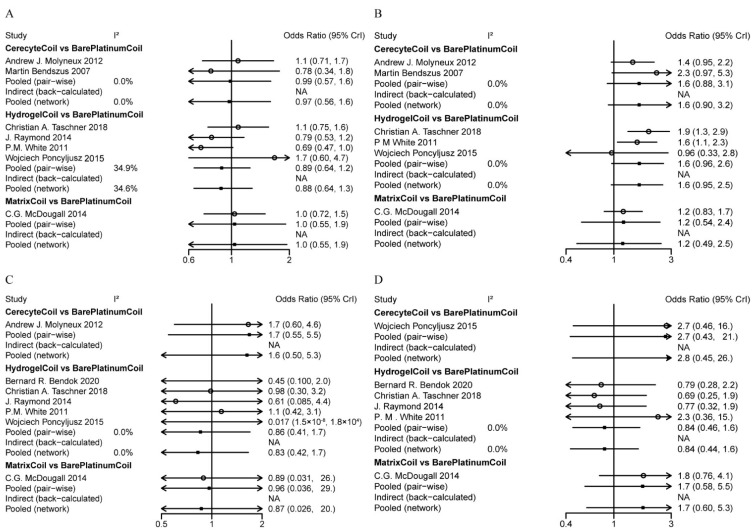
Forest plots for the heterogeneity of efficacy and safety indicators (Martin et al. [18], Andrew et al. [19], White et al. [22], McDougall et al. [24], Raymond et al. [20], Wojciech et al. [21], Christian et al. [23], Bernard et al. [10]). (**A**) Percentage of postoperative patients with modified Raymond scale grade I; (**B**) Percentage of patients with modified Raymond scale grade I at follow-up; (**C**) Rates of intraoperative aneurysm rupture or re-rupture; (**D**) Mortality rate.

To confirm the reasonableness of the process of combining data from different studies and meta-analysis. We performed heterogeneity analysis for each included indicator, and the analysis of direct and indirect evidence is presented in Figure 5 and Supplementary Material Figure S5. By analyzing the heterogeneity of the studies overall, we found the following data: The overall heterogeneity in the percentage of the population with modified Raymond scale grade II at follow-up (*I*^2^ = 86.3073%) was greater than 50%. A random-effects model was performed for meta-analysis of this indicator according to the previously described methods. For the other data, the overall *I*^2^ was 0% except for the rate of aneurysm rupture or re-rupture (*I*^2^ = 32.7693%), and all indicators above were analyzed using fixed-effects models. No consistency test was performed and was not required because none of the data structures had indirect evidence.

## 4. Discussion

Intracranial aneurysms are intracranial arterial wall lesions that tend to occur in middle-aged women [1]. Current treatments, including surgery and endovascular embolization, aim at isolating the aneurysm from circulation [27]. Endovascular embolization is a widely performed treatment for intracranial aneurysms. This technique isolates the aneurysm from the intracranial circulation by filling the aneurysm with coils [28]. It has the advantages of being minimally invasive and having a high embolization rate and short post-operative recovery time [29]. In this study, we analyzed the embolic effects and safety of different coils, providing evidence-based medical advice on the choice of coils for endovascular interventions.

In terms of effectiveness, the modified Raymond scale is a widely accepted system for assessing the grade of aneurysm occlusion and is divided into three grades, with the modified Raymond scale grade I being defined as complete occlusion, grade II as residual neck, and grade III as residual aneurysm [30]. We compared the percentage of people with different grades of the modified Raymond scale at post-operative and follow-up stages. At the post-operative stage, there was no significant difference in the proportion of patients to any of the three grades of the modified Raymond scale when four coils were paired and compared in the network. This result is thought to be due to fact that the bioactive coils are bare platinum coils modified with a different bioactive material, whose small differences in platinum content, stiffness, and mechanical properties do not change the surgical outcome significantly [31,32]. In contrast, we hypothesize that the operator’s surgical technique has a greater impact on the embolic effect evaluated immediately after the procedure. At follow-up, we found that the hydrogel coils exhibited a statistically significantly higher percentage of patients with a modified Raymond scale grade I, compared to the bare platinum coil group. This finding is consistent with the SCURA values and rank ranking. This result is very favorable for the use of bioactive coils compared to the no statistical difference at immediate postoperative evaluation. Unfortunately, other pairwise comparisons between coils were not able to find statistically significant differences.

Regarding the different statistical results exhibited by the hydrogel coils in the above two time periods, we believe that they should be interpreted in two ways. On the one hand when it comes to postoperative assessments, several previous studies [33,34] have shown that the packing density of aneurysms treated with hydrogel coils was significantly higher, between 36% and 68%, than that of aneurysms treated with bare platinum coils [35]. In addition, hydrogel coils are more evenly distributed in the aneurysm sac, particularly in the neck of the aneurysm, which may be more helpful in achieving better occlusion rates [36]. However, these place greater demands on the operator’s surgical skills, and the effect of operator manipulation on coil outcomes is unavoidable and significantly confounding in the literature we included. Our analysis of the percentage of patients at all grades of the modified Raymond Scale for immediate post-operative assessments failed to result in a difference based on the available data and was, to some degree, influenced by this. On the other hand, at follow-up, changes occurred and were found to be statistically significant in hydrogel coils compared to bare platinum coils, and the lack of human involvement in this change process was considered more credible for the results. Previous studies have shown that aneurysm embolization is often accompanied by changes in the biological properties of the aneurysm wall [3]. Hydrogel coils are thought to have a better biological response, with more endothelial deposition in the neck, more cellular response, and thicker neointima formation, leading to a better aneurysm occlusion rate [35]. This process takes some time to occur, so it is not confusing to find statistical differences at follow-up.

In clinical applications, coil selection should focus not only on effectiveness but also on its safety implications. In order to explore which coils are a relatively safe choice for clinical application, the safety indicators chosen for the study included the rate of aneurysm rupture or re-rupture and mortality, but no statistical differences were found between the four aneurysms for the two indicators of safety. This suggests that bioactive coils have no risks in the increase probability of these safety indicators.

However, there are still some shortcomings in our study, such as: (1) The small number of studies included in this study of matrix coils, with only one RCT, needs to be further investigated as to whether this introduces error to the results. (2) The small sample sizes of some RCTs, such as articles by Martin Bendszus et al., 2007 [19], and Wojciech Poncyljusz et al., 2015 [22], may have had an impact on the results of the analysis. (3) While most RCTs had a follow-up time equal to or more than 6 months, J. Raymond et al., 2014 [21] only had 1 month, which does not give a better indication of the efficiency and safety of the treatment and may affect the credibility of the conclusions. (4) Some of the RCTs included in the study had missing or incomplete follow-up results, which could potentially affect the results. (5) For hydrogel coils, the differences between the two generations of materials were not analyzed in subgroups due to too few articles suitable for inclusion and minor differences in the envelope materials. (6) This network meta-analysis was not registered.

In summary, we conducted a network meta-analysis of four coils, namely cerecyte coils, hydrogel coils, matrix coils, and bare platinum coils. The effectiveness and safety of the different coils were compared by the statistical analysis of eight RCTs. Compared with other coils, hydrogel coils had a higher rate of complete embolization at follow-up, while there was no difference in safety between hydrogel and other coils. This network meta-analysis provides a theoretical reference for the clinical treatment of aneurysms, but due to the limitations mentioned above, more clinical studies are needed to validate this conclusion. As bioactive coils continue to be refined, they will become increasingly advantageous in the treatment of aneurysms.

## Figures and Tables

**Table 1:** Characteristics of the Included Studies and Outcome Events.

Study	Centers Included	Treatment Group, (No. of Participants)	Female (%)	Mean Age (SD) (Year)	Follow-Up Time	Neck Size (SD)	Ruptured/Unruptured	Outcome Events
Martin et al. [18]	1	CCC (55) vs. BPC (55)	CCC 32.7% BPC 38.1%	CCC 48 (11.0) BPC 51 (10.0)	6 months	CCC 3.3 (0.8) BPC 3.1 (1.2)	CCC 0/55 BPC 0/55	a,b,c,d,e,f
Andrew et al. [19]	23	CCC (249) vs. BPC (251)	CCC 70.0% BPC 72.4%	CCC 50.2 (10.3) BPC 51.1 (10.1)	6 months	NA	CCC 116/133 BPC 120/131	a,b,c,d,e,f,g
White et al. [22]	24	HEC (249) vs. BPC (250)	HEC 70.7% BPC 69.6%	<45 year 80/78 46–55 year 68/68 >55 year 101/101 *	18 months	NA	HEC 139/110 BPC 138/112	a,b,c,d,e,f,g,h
McDougall et al. [24]	1	MAC (311) vs. BPC (315)	MAC 73.6% BPC 67.0%	MAC 55.7 (11.6) BPC 54.4 (13.2)	15 months	MAC 3.6 (1.5) BPC 3.7 (1.6)	MAC 109/202 BPC 119/196	a,b,c,d,e,f,g,h
Raymond et al. [20]	25	HEC (225) vs. BPC (222)	HEC 73.3% BPC 69.4%	HEC 57 (11.0) BPC 58 (12.0)	1 month	HEC 4.9 (2.2) BPC 4.6 (2.1)	HEC 40/185 BPC 39/183	a,b,c,g,h
Wojciech et al. [21]	1	HEC (50) vs. BPC (46)	HEC 62.0% BPC 65.2%	HEC 49.6 (10.8) BPC 52.2 (8.7)	12 months	HEC 4.4 (2.3) BPC 4.5 (2.3)	HEC 0/50 BPC 0/46	a,b,c,d,e,f,g,h
Christian et al. [23]	22	HEC (243) vs. BPC (241)	HEC 71.0% BPC 67.0%	HEC 52.9 (12.6) BPC 54.1 (11.8)	18 months	HEC 3.5 (1.3) BPC 3.6 (1.3)	HEC 103/140 BPC 105/136	a,b,c,d,e,f.g,h
Bernard et al. [10]	46	HEC (297) vs. BPC (303)	HEC 80.1% BPC 77.9%	HEC 56.5 (11.5) BPC 56.9 (10.3)	12 months	HEC 3.2 (1.4) BPC 3.0 (1.4)	HEC 76/216 BPC 93/208	g,h

CCC: Cerecyte Coil; BPC: Bare Platinum Coil; HEC: Hydrogel Coil; MAC: Matrix Coil; N/A: not applicable; a—Percentage of postoperative modified Raymond scale grade I patients; b—Percentage of postoperative modified Raymond scale grade II patients; c—Percentage of postoperative modified Raymond scale grade III patients; d—Percentage of patients with modified Raymond scale grade I at follow-up; e—Percentage of patients with modified Raymond scale grade II at follow-up; f—Percentage of patients with modified Raymond scale grade III at follow-up; g—Rates of intraoperative aneurysm rupture or re-rupture; h—Mortality rate. * The ratio of HEC to BPC in different age groups or different aneurysm size layers.

## Data Availability

All data generated or analyzed during this study are included in this published article and its supplementary information files.

## Supplementary Materials

The following supporting information can be downloaded at: https://www.mdpi.com/article/10.3390/brainsci12081062/s1, Figure S1: Network of efficacy and safety indicators. Figure S2: Trace diagram of Markov Monte Carlo method. Figure S3: League tables for the outcomes of efficacy and safety generated using fixed or random effect models. Figure S4: Probability ranks for outcomes of the efficacy and safety generated using fixed or random effect models. Figure S5: Forest plots for the heterogeneity of efficacy and safety indicators.

## Abbreviations

RCT: randomized controlled trial; OR: odds ratio; SUCRA: surface under the cumulative ranking curve; CI: confidence interval; PGLA: polyglycolic/polylactic acid.
