# Supplementary material for: Efficacy and Safety of Different Bioactive Coils in Intracranial Aneurysm Interventional Treatment, a Systematic Review and Bayesian Network Meta-Analysis
Source: Brain Sci. 2022 Aug 10;12(8):1062. doi: 10.3390/brainsci12081062 (PMC9405728; doi:10.3390/brainsci12081062)

Supplement I

Figure S1 | Network of efficacy and safety indicators. (a) Percentage of postoperative modified Raymond Scale grade I patients. (b) Percentage of postoperative modified Raymond Scale grade II patients. (c) Percentage of postoperative modified Raymond Scale grade III patients. (d) Percentage of patients with modified Raymond Scale grade I at follow-up. (e) Percentage of patients with modified Raymond Scale grade II at follow-up. (f) Percentage of patients with modified Raymond Scale grade III at follow-up. (g) Rates of intraoperative aneurysm rupture or re-rupture. (h) Mortality rate.

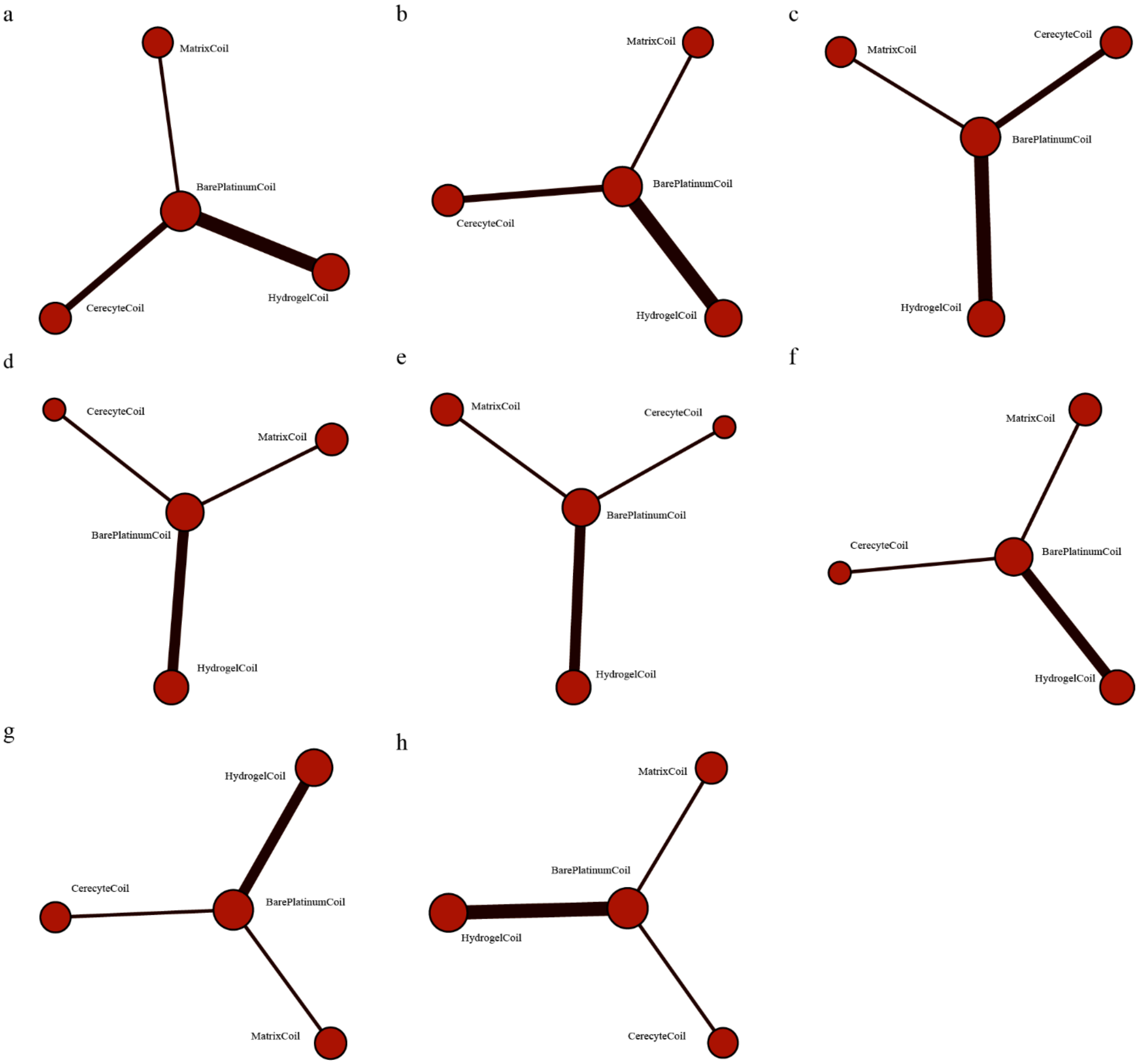

Supplement II

Figure S2 | Trace diagram of Markov Monte Carlo method. (a) Percentage of postoperative modified Raymond Scale grade I patients. (b) Percentage of postoperative modified Raymond Scale grade II patients. (c) Percentage of postoperative modified Raymond Scale grade III patients. (d) Percentage of patients with modified Raymond Scale grade I at follow-up. (e) Percentage of patients with modified Raymond Scale grade II at follow-up. (f) Percentage of patients with modified Raymond Scale grade III at follow-up. (g) Rates of intraoperative aneurysm rupture or re-rupture. (h) Mortality rate.

a

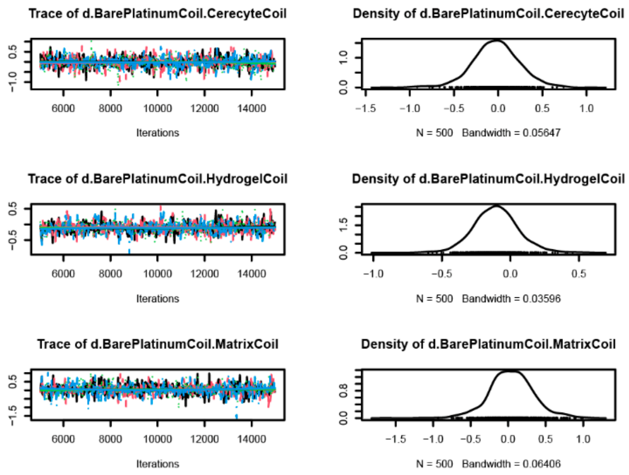

b

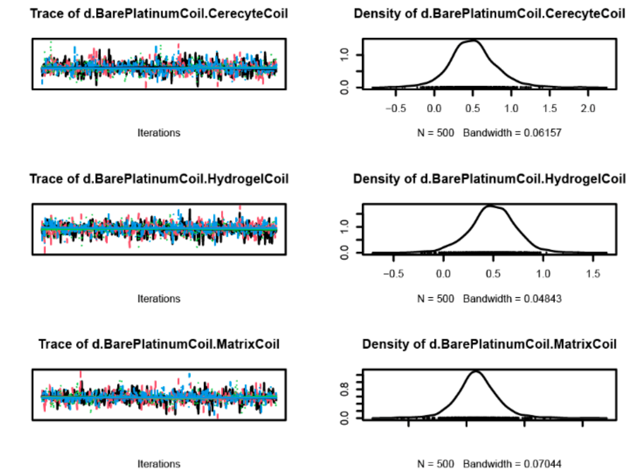

c

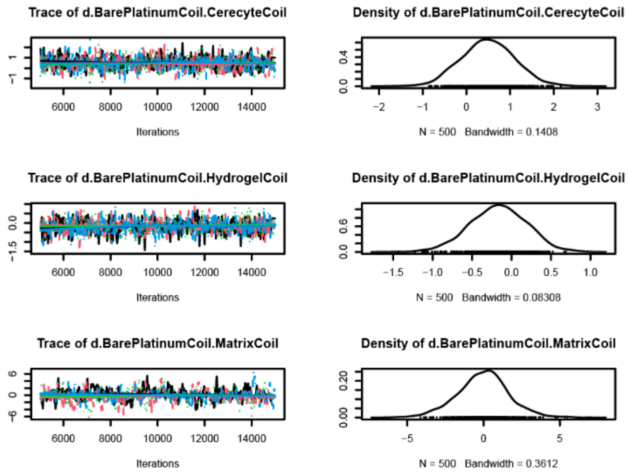

d

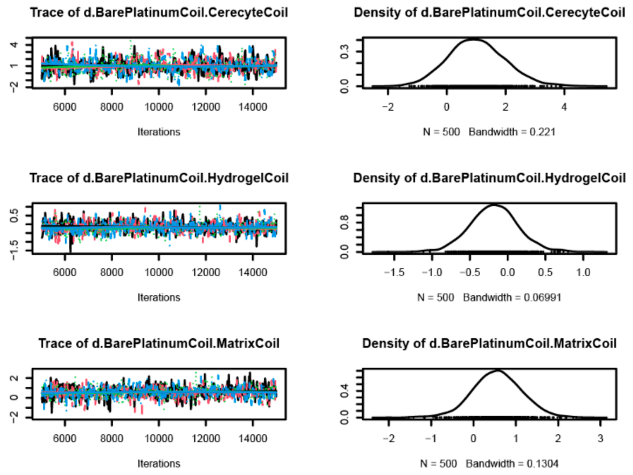

e

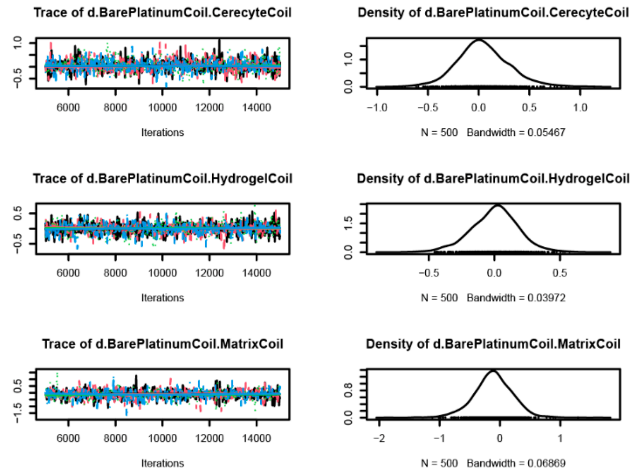
Trace of d.BarePlatinumCoil.CerecyteCoil
Iterations
Density of d.BarePlatinumCoil.CerecyteCoil
N = 500 Bandwidth = 0.05467
Trace of d.BarePlatinumCoil.HydrogelCoil
Iterations
Density of d.BarePlatinumCoil.HydrogelCoil
N = 500 Bandwidth = 0.03972
Trace of d.BarePlatinumCoil.MatrixCoil
Iterations
Density of d.BarePlatinumCoil.MatrixCoil
N = 500 Bandwidth = 0.06869

f

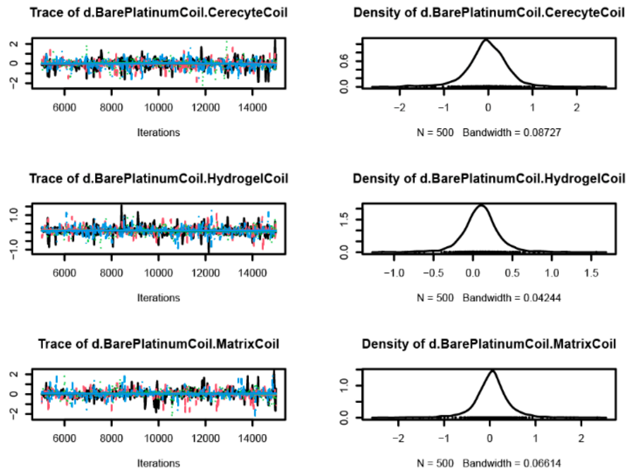
Trace of d.BarePlatinumCoil.CerecyteCoil
Iterations
Density of d.BarePlatinumCoil.CerecyteCoil
N = 500 Bandwidth = 0.08727
Trace of d.BarePlatinumCoil.HydrogelCoil
Iterations
Density of d.BarePlatinumCoil.HydrogelCoil
N = 500 Bandwidth = 0.04244
Trace of d.BarePlatinumCoil.MatrixCoil
Iterations
Density of d.BarePlatinumCoil.MatrixCoil
N = 500 Bandwidth = 0.06614

g

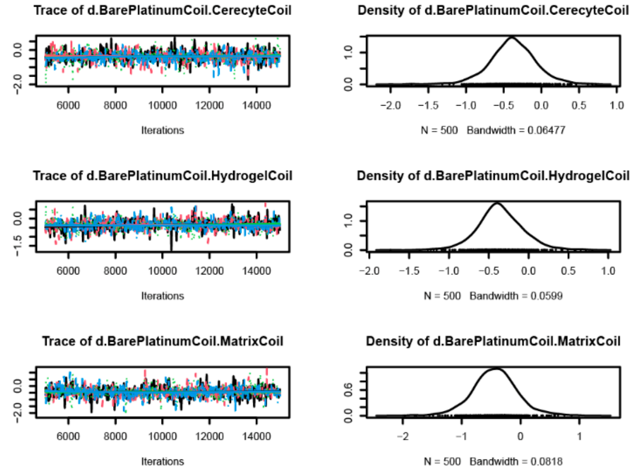
Trace of d.BarePlatinumCoil.CerecyteCoil
Iterations
Density of d.BarePlatinumCoil.CerecyteCoil
N = 500 Bandwidth = 0.06477
Trace of d.BarePlatinumCoil.HydrogelCoil
Iterations
Density of d.BarePlatinumCoil.HydrogelCoil
N = 500 Bandwidth = 0.0599
Trace of d.BarePlatinumCoil.MatrixCoil
Iterations
Density of d.BarePlatinumCoil.MatrixCoil
N = 500 Bandwidth = 0.0818

h

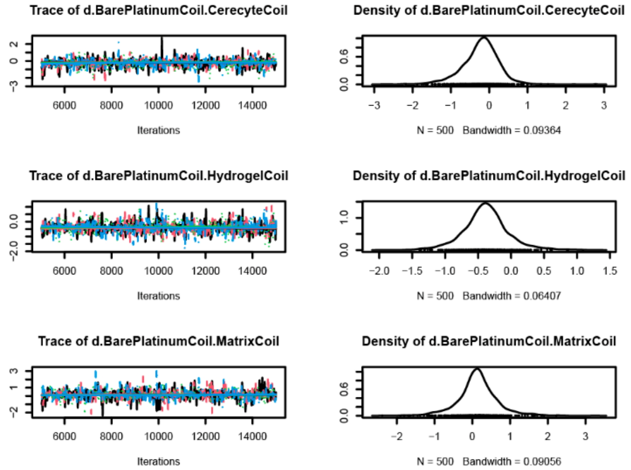
Trace of d.BarePlatinumCoil.CerecyteCoil
Iterations
Density of d.BarePlatinumCoil.CerecyteCoil
N = 500 Bandwidth = 0.09364
Trace of d.BarePlatinumCoil.HydrogelCoil
Iterations
Density of d.BarePlatinumCoil.HydrogelCoil
N = 500 Bandwidth = 0.06407
Trace of d.BarePlatinumCoil.MatrixCoil
Iterations
Density of d.BarePlatinumCoil.MatrixCoil
N = 500 Bandwidth = 0.09056

Supplement III

Figure S3 | League tables for the outcomes of efficacy and safety generated using fixed or random effect models. (a) Percentage of postoperative modified Raymond Scale grade II patients. (b) Percentage of postoperative modified Raymond Scale grade III patients. (c) Percentage of patients with modified Raymond Scale grade II at follow-up. (d) Percentage of patients with modified Raymond Scale grade III at follow-up.

a

	BarePlatinumCoil	CerecyteCoil	HydrogelCoil	MatrixCoil
BarePlatinumCoil	SUCRA 0.5262			
CerecyteCoil	-0.028 (-0.6041, 0.4445)	SUCRA 0.5758		
HydrogelCoil	-0.0100 (-0.3656, 0.3855)	0.0251 (-0.5478, 0.7264)	SUCRA 0.5483	
MatrixCoil	0.1111 (-0.5121, 0.7962)	0.1435 (-0.6436, 1.0666)	0.1194 (-0.6464, 0.8886)	SUCRA 0.3497

b

	BarePlatinumCoil	CerecyteCoil	HydrogelCoil	MatrixCoil
BarePlatinumCoil	SUCRA 0.4178			
CerecyteCoil	0.0169 (-0.8398, 1.0643)	SUCRA 0.4517		
HydrogelCoil	-0.0989 (-0.603, 0.3858)	-0.0923 (-1.3164, 0.8797)	SUCRA 0.6183	
MatrixCoil	-0.0458 (-0.8816, 0.9199)	-0.0303 (-1.4849, 1.2362)	0.0453 (-0.9234, 1.1631)	SUCRA 0.5122

c

	BarePlatinumCoil	CerecyteCoil	HydrogelCoil	MatrixCoil
BarePlatinumCoil	SUCRA 0.8938			
CerecyteCoil	0.3789 (-0.2590, 1.0276)	SUCRA 0.3810		
HydrogelCoil	0.3689 (-0.2637, 0.9211)	-0.0123 (-0.9408, 0.8189)	SUCRA 0.3983	
MatrixCoil	0.4338 (-0.4082, 1.2243)	0.0565 (-1.0335, 1.0870)	0.0840 (-0.9719, 1.0963)	SUCRA 0.3268

d

	BarePlatinumCoil	CerecyteCoil	HydrogelCoil	MatrixCoil
BarePlatinumCoil	SUCRA 0.6438			
CerecyteCoil	0.1859 (-0.6461, 1.3321)	SUCRA 0.4140		
HydrogelCoil	0.3878 (-0.3854, 1.1028)	0.2107 (-1.2048, 1.1821)	SUCRA 0.2028	
MatrixCoil	-0.1396 (-1.3690, 1.0334)	-0.3147 (-1.9670, 1.0083)	-0.5279 (-1.905, 0.8692)	SUCRA 0.7393

No Significance	Column vs Row Log OR (95% CI)	Significance

Supplement IV

Figure S4 | Probability ranks for outcomes of the efficacy and safety generated using fixed or random effect models. (a) Percentage of postoperative modified Raymond Scale grade II patients. (b) Percentage of postoperative modified Raymond Scale grade III patients. (c) Percentage of patients with modified Raymond Scale grade II at follow-up. (d) Percentage of patients with modified Raymond Scale grade III at follow-up.

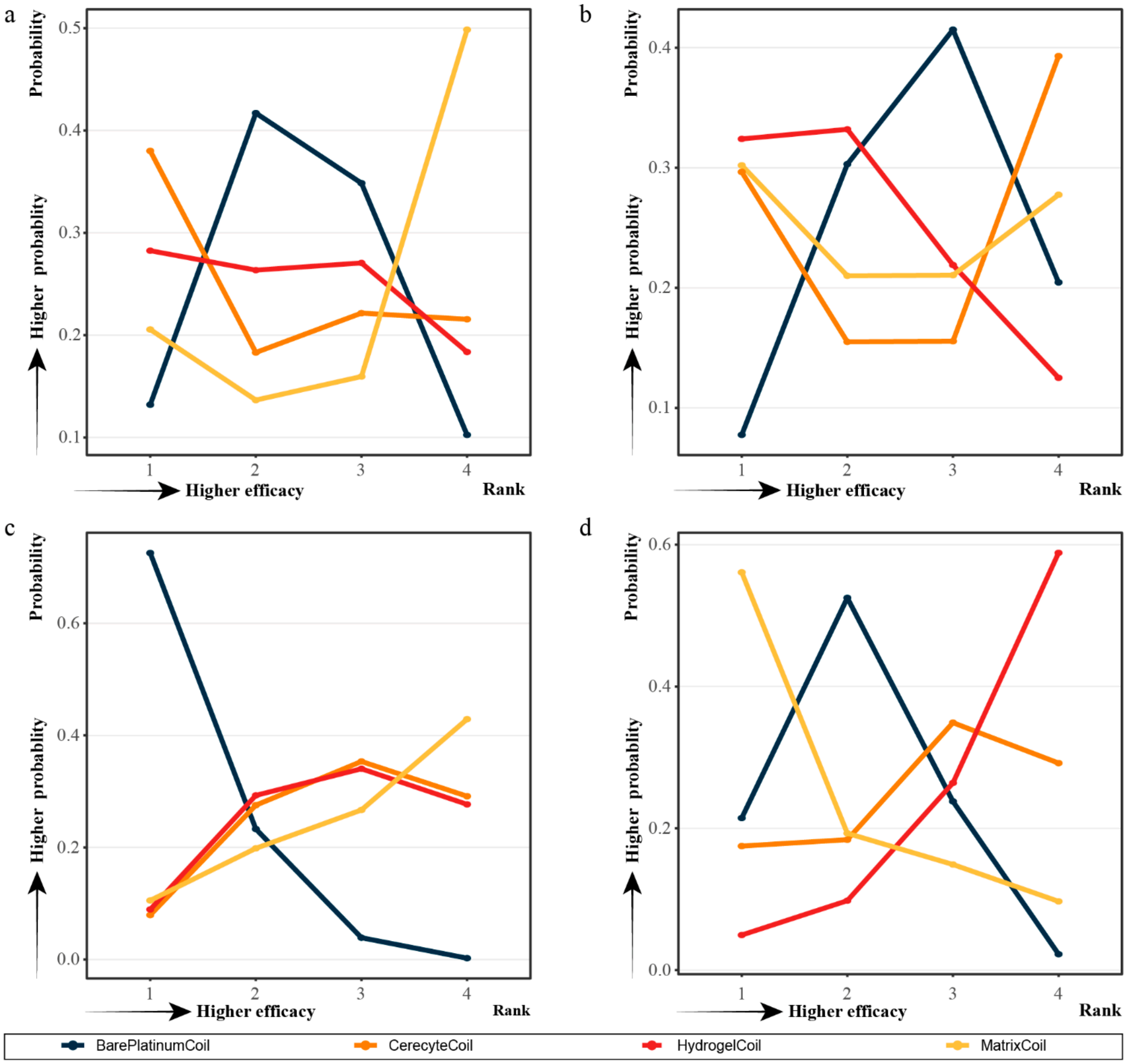

Supplement V

Figure S5 | Forest plots for the heterogeneity of efficacy and safety indicators. (a) Percentage of postoperative modified Raymond Scale grade II patients. (b) Percentage of postoperative modified Raymond Scale grade III patients. (c) Percentage of patients with modified Raymond Scale grade II at follow-up. (d) Percentage of patients with modified Raymond Scale grade III at follow-up.

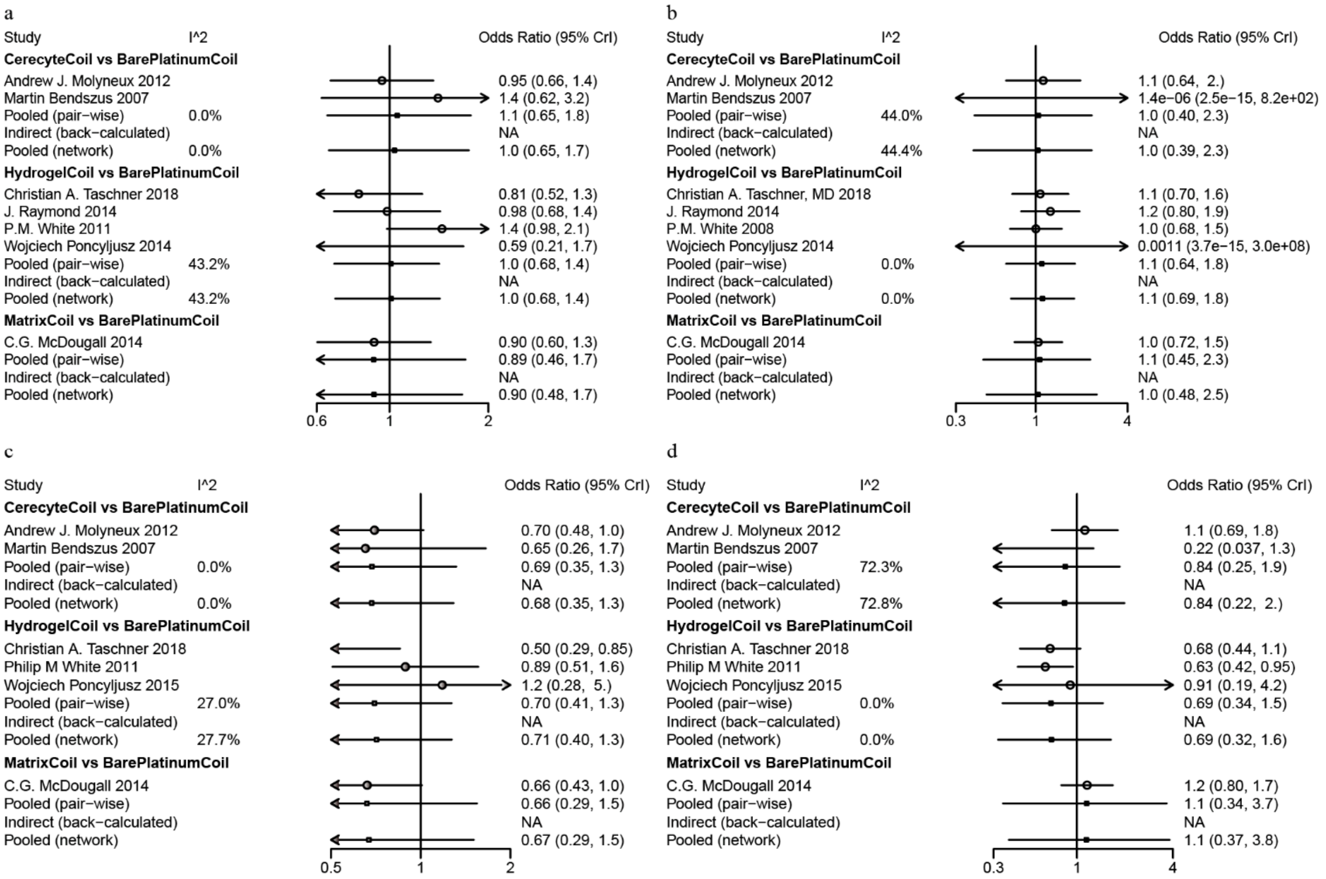

Supplement: Supplementary file 1 [file brainsci-12-01062-s001.zip › brainsci-1805040-supplementary.pdf]
